# Observing atomic layer electrodeposition on single nanocrystals surface by dark field spectroscopy

**DOI:** 10.1038/s41467-020-16405-3

**Published:** 2020-05-20

**Authors:** Shu Hu, Jun Yi, Yue-Jiao Zhang, Kai-Qiang Lin, Bi-Ju Liu, Liang Chen, Chao Zhan, Zhi-Chao Lei, Juan-Juan Sun, Cheng Zong, Jian-Feng Li, Bin Ren

**Affiliations:** State Key Laboratory of Physical Chemistry of Solid Surfaces, Collaborative Innovation Center of Chemistry for Energy Materials (i-ChEM), Department of Chemistry, College of Chemistry and Chemical Engineering, Xiamen University, 361005 Xiamen, China

**Keywords:** Electrochemistry, Materials chemistry, Physical chemistry, Optical spectroscopy, Surface chemistry

## Abstract

Underpotential deposition offers a predominant way to tailor the electronic structure of the catalytic surface at the atomic level, which is key to engineering materials with a high activity for (electro)catalysis. However, it remains challenging to precisely control and directly probe the underpotential deposition of a (sub)monolayer of atoms on nanoparticle surfaces. In this work, we in situ observe silver electrodeposited on gold nanocrystals surface from sub-monolayer to one monolayer by designing a highly sensitive electrochemical dark field scattering setup. The spectral variation is used to reconstruct the optical “cyclic voltammogram” of every single nanocrystal for understanding the underpotential deposition process on nanocrystals, which cannot be achieved by any other methods but are essential for creating novel nanomaterials.

## Abstract (web summary)

Underpotential deposition (UPD) is important to modify the surface properties of nanocrystals. Here, the authors show the application of in situ electrochemical dark field spectroscopy in identifying the UPD processes of silver on different facets of gold nanocrystals at the single nanoparticle level.

## Introduction

Engineering nanomaterials of high catalytic activity and stability with a low cost has been an eternal subject for electrocatalysis^1–4^. Altering the electronic structure of atoms at the topmost layer where the reaction occurs can be the most efficient way for this subject. However, it remains challenging to find a technique that can precisely tune the surface atoms at the atomic level without introducing strong binding ligands. Underpotential deposition (UPD) provides a predominant way to accomplish such a purpose, since it enables controllable deposition of foreign metal atoms at the submonolayer level to create a clean surface with unique physical and chemical properties^5–8^. The UPD strategy has been intensely applied to assist wet chemical synthesis of nanoparticles (NP) with a controllable shape and size or modify over the existing NPs to create novel catalysts^9–13^. However, the practice is essentially empirical since a clear understanding of the UPD mechanism is still limited to bulk single crystals even after decades’ investigation^6^. It is still a great challenge to directly study the UPD process on individual NPs with a complex morphology, adsorbed species, and unique surface energy, even for in situ transmission electron microscopy (TEM)^14^ and scanning probe microscopies (SPM)^15^.

Here, we develop a highly sensitive electrochemical dark field scattering (EC-DFS) setup for in situ monitoring the UPD process of Ag on Au nanocrystals (NCs). It enables identification of small spectral variations induced by the deposition of submonolayer atoms in the electrochemical environment. More importantly, we reconstruct the optical “cyclic voltammogram (CV)” from the spectral variation, which not only precisely gives the potential and band width of UPD process of different individual NCs but also displays the energy difference of different facets on a NC. This work provides unprecedented information for understanding the UPD process on NCs, and allows the control of the UPD process at the single NP level that are of both fundamental and technological importance for electrocatalysis.

## Results

### The setup for EC-DFS technique

**Fig. 1:**
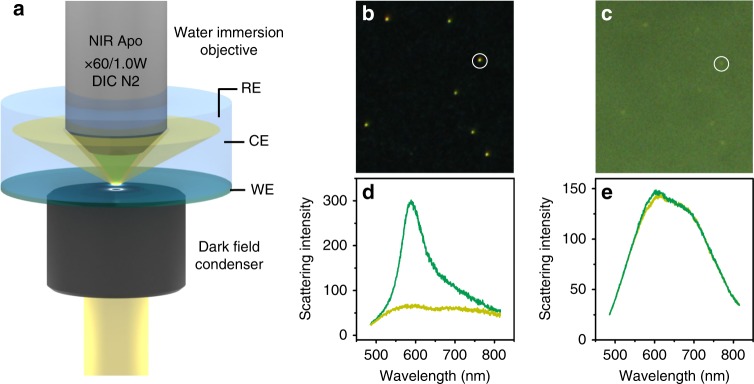
A novel setup for the electrochemical dark field scattering technique. **a** A schematic of the dark field microscopy and spectroscopy on the basis of a water immersion objective (WE: working electrode, CE: counter electrode, RE: reference electrode). Dark field image (**b**) and spectra (**d**) obtained on the new setup. Dark field image (**c**) and spectra (**e**) obtained on the conventional setup. The exposure times for spectra in **d** and **e** are 1 and 10 s (normalized to 1 s), respectively.

We designed a highly sensitive EC-DFS setup for more challenging systems with weak signals while maintaining the accurate and stable potential control. As depicted in Fig. 1a, an oil immersion dark field condenser with a high NA of 1.4–1.6 was utilized to illuminate an ITO electrode from the bottom, and a water immersion objective (with NA of 1.0) covered with a polyethylene film was directly dipped into the electrolyte. Such a working mode can not only effectively suppress the mismatch of the refractive index in the optical path but also significantly increase the excitation and collection efficiency. Consequently, the sensitivity of the EC-DFS technique can be dramatically improved (detailed descriptions of the setup and novelty compared with the conventional setup can be found in Supplementary Note 1). Figure 1b, c compare the dark field images of a sample in the same area obtained by the new and conventional setups, respectively. It can be found that the octahedral Au NCs (bright spots) in the image of Fig. 1b exhibit much better visibility even with a lower exposure time and gain compared with Fig. 1c. The improvement in the detection sensitivity is clear by comparing the relative intensity of the scattering spectra from a single NC and the background spectra of the substrate in the two setups (Fig. 1d, e). The spectrum acquired with a 1 s acquisition time by the new setup exhibits not only much higher signal but also lower background than that by the conventional setup even with 10 s acquisition time (normalized to 1 s), resulting in a 50-fold improvement of the signal to background ratio (see Supplementary Fig. 2). Such a great improvement enables the detection of the spherical Au NPs with a diameter as low as 10–15 nm (compared with that of 50 nm in the literature^16^) without using a special light source or equipment, as we demonstrated in Supplementary Fig. 3. The high sensitivity is also essential for identifying the small spectral variation and tracking dynamic processes during the electrochemical sensing as well as other applications, such as biosensing and single NP catalysis.

### Ag UPD on single octahedral Au NCs

**Fig. 2:**
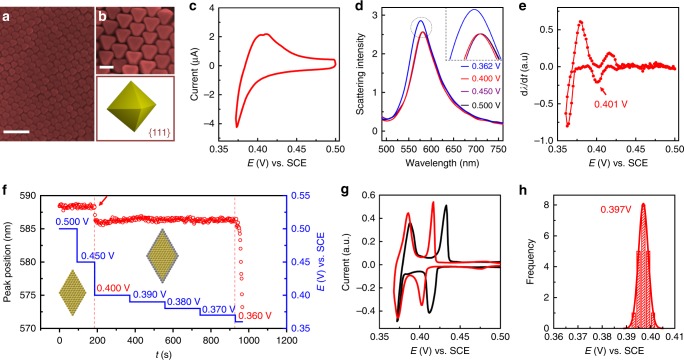
Study of Ag electrodeposit on the octahedral Au nanocrystals surface. SEM images of **a** large and **b** (top) small scale along with **b** (bottom) structural schematic of Au nanooctahedrons to show their highly ordered geometry on the electrode surface and the structural details. **c** A cyclic voltammogram (CV) of Ag underpotential deposition (UPD) on a Au nanooctahedrons-coated glassy carbon electrode in the electrolyte of 1 mM Ag_2_SO_4_ and 0.05 M H_2_SO_4_. **d** The potential-dependent scattering spectra of a single Au nanooctahedron. The inset is the zoom-in spectra showing the details of spectral variation. **e** The optical “CV” of a single Au nanooctahedron reconstructed using the peak position of the scattering spectra as a function of the applied potential. **f** The time-dependent peak position change of the scattering spectra of a single Au nanooctahedron under different potential controls (from 0.500 to 0.400 V with a step of 50 mV and from 0.400 to 0.360 V with a step of 10 mV). **g** CVs for the Ag deposition on a Au (111) electrode before (black curve) and after (red curve) the adsorption of the surfactant, CTAB. **h** The peak potential statistics of UPD peaks obtained from the optical “CV”s of 20 individual Au nanooctahedrons. The scale bars are 200 nm in **a** and 50 nm in **b**.

Ag UPD on Au is a widely used route for tailoring the electrocatalytic activity and assisting the synthesis of nanomaterials^3,17–19^. Although electrochemical scanning tunneling microscopy (EC-STM) has been widely used to characterize the UPD process on the bulk single crystal electrode surface^20^, it is technically difficult to characterize UPD on NPs surfaces. We used the cyclic voltammetry to investigate the UPD process. The CV curve of Ag deposition on a Au nanooctahedrons-coated (with exposed Au (111) facet) glassy carbon electrode (Fig. 2a, b) is shown in Fig. 2c. Unfortunately, we could not identify any apparent UPD peaks from the CV curve, even when the NPs were coated on the electrode surface in a uniform closely packed structure and had an extreme narrow size distribution (Fig. 2a). The main reason for this can be the NPs with a high coverage compresses the interparticle electrolyte into an extreme thin layer. The silver ions within the nanogap quickly deplete during the deposition, which leads to the continuous negative shift of UPD peaks. The variation of the gap distances between NPs further results in a broad distribution of the UPD potential and the broadened UPD peaks. However, such an effect is not as significant in the silver dissolution process as that in the UPD process since the silver atoms are already on the NPs surface. Consequently, the corresponding dissolution peak of UPD can still be well distinguished from OPD, although is already broader than that on the single crystal surface (see Fig. 2c). Single particle electrochemistry can avoid such an effect and provide intrinsic electrochemical features of individual NCs^21–26^. Therefore, we used the above EC-DFS technique to track the Ag UPD process at the single NP level (Fig. 1). An ITO electrode with a low density of Au NCs was used as the working electrode. The potential of the electrode was scanned from 0.500 to 0.362 V using cyclic voltammetry (1 mV/s), and the potential-dependent scattering spectra (see Fig. 2d) from a single NC were simultaneously acquired. For clarity, the peak position and intensity are plotted as a function of potential (Supplementary Fig. 4a). The peak position blue shifts from 0.413 V and remains constant at potentials between 0.390 and 0.370 V. A more significant blue shift is observed at the potentials lower than 0.370 V along with the increase of the spectral intensity. We confirm such spectral shifts are induced by the Ag deposition from our control experiments in solutions with electrolyte only and a lower concentration of Ag ion (Supplementary Note 2). If we take the first-order derivative of the spectral shift with time and plot it with potential, we obtain a curve shown in Fig. 2e with a similar shape to the CV, which is called optical “CV”. The curve consists of two obvious deposition peaks that may correspond to the UPD and OPD processes on the single NC. We performed the potential step experiment while simultaneously acquiring the scattering spectra to verify these two deposition processes. The spectra almost remain the same at 0.500 and 0.450 V (Fig. 2f). Whereas, once the potential reaches the first deposition peak (see Fig. 2e) of 0.400 V, the peak blue shifts. The shift stops in just a few seconds. Afterwards, the spectra remain almost the same with the further negative movement of the potential. Such a phenomenon agrees well with the feature of the UPD process, i.e., once a full monolayer has been achieved, no further deposition can occur. Moreover, the scanning electron microscopy (SEM) images show that the morphology of Au nanooctahedrons after UPD potential remains unchanged (see Supplementary Note 3), which excludes the possibilities of heterogeneous selective growth of Ag on the vertex or morphological change of NCs. When the potential reaches the second deposition process (Fig. 2e) of 0.360 V, the spectra show a fast and continuous blue shift, which agrees with the feature of the overpotential deposition (OPD) process. More direct evidence can be seen from the CV of Ag deposition on the Au (111) single crystal electrode (Fig. 2g). The CV of a clean Au (111) electrode (Fig. 2g, black curve) clearly distinguishes the UPD process from the bulk deposition by showing a current peak at 0.412 V, in good agreement with the literatures^20^. However, the peak potential is 12 mV more positive than that of the first deposition peak in Fig. 2e, which may indicate the lowering of the surface energy of the facet due to the presence of the surfactant on NCs. To verify this effect, we modified the Au (111) electrode surface with the same surfactant, then repeated the CV measurements. As shown in Fig. 2g (red curve), the UPD potential shifts from 0.412 to 0.402 V after the modification with surfactant, agreeing with the peak potential (0.401 V) in Fig. 2e for single NC. The result convincingly demonstrates that the first (0.401 V) and second (0.362 V) deposition peaks in the optical “CV” of a single Au nanooctahedron correspond to the UPD and OPD processes, respectively. It is interesting to see that the peak width of the UPD peaks of single Au nanooctahedron is as narrow as that of the Au (111) electrode, indicating that the facet quality of the fresh NCs is as good as that of the single crystal electrode. In Fig. 2h, we present a histogram of the UPD peak positions of the “CV”s from the 20 individual NCs, which shows a distribution of the deposition potential from 0.393 to 0.401 V with a peak width of 5 mV. This result indicates different surface states of different NCs, which cannot be observed in ensemble systems. We believe the UPD potential distribution is mainly induced by the variation in the size of the NC, which results in the small surface energy difference of the facets (see Supplementary Note 4). Consequently, one can observe the UPD potential follows a similar distribution to the size (see Supplementary Fig. 8).

### Ag UPD on single cubic Au NCs surface

**Fig. 3:**
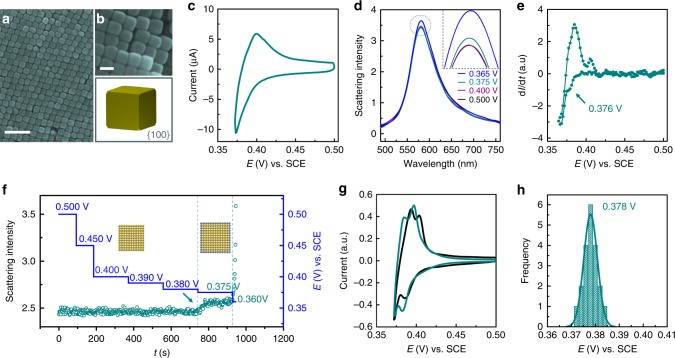
The electrodeposition of Ag on the cubic Au nanocrystals surface. SEM images of **a** large and **b** (top) small-scale reveal the highly ordered geometry of Au nanocubes on the electrode along with **b** (bottom) structural schematic of the Au nanocube. **c** The CV of Ag UPD on the Au nanocubes-coated electrode in the electrolyte of 1 mM Ag_2_SO_4_ and 0.05 M H_2_SO_4_. **d** The scattering spectra of single Au nanocube under different potentials. The inset shows the zoom-in spectra to clearly present the spectral variations. **e** The reconstructed optical “CV” of single Au nanocube, showing two different deposition processes. **f** The time-dependent peak intensity change of the scattering spectra of single Au nanocube under different potential controls (from 0.500 to 0.400 V with a step of 50 mV, from 0.400 to 0.380 V with a step of 10 mV and from 0.380 to 0.360 V with a step of 15 mV). **g** The CVs of Ag deposition on a Au (100) electrode before (black curve) and after (dark cyan curve) the adsorption of the surfactants, CTAB. **h** The peak potential statistics of UPD bands obtained from the optical “CV”s of 20 individual Au nanocubes. The scale bars are 200 nm in **a** and 50 nm in **b**.

To demonstrate the generality of our method, we performed the same experiments on cubic Au NCs enclosed by {100} facets (Fig. 3b). Figure 3c shows the CV of Ag deposition on a Au nanocubes-coated electrode. Similar to Au nanooctahedrons (Fig. 2c), the UPD peak is still not discernible due to the average effect. The change in the single NC scattering spectra can be clearly observed after Ag deposition (Supplementary Note 2). Compared with the Au nanooctahedrons, we found the peak intensity change occurs prior to the spectral shift, which indicates that the peak intensity is more sensitive to the Ag deposition than the peak position in the case of Au nanocubes (in Supplementary Fig. 4b). The scattering spectra of nanooctahedrons and nanocubes respond differently upon the silver deposition. We believe it is the aspect ratio change upon deposition that determines the different responses in nanooctahedron and nanocube systems (see Supplementary Fig. 9). For example, for the nanooctahedron, the aspect ratio decreases after the silver deposition, which will induce a significant spectral shift, similar to nanorod. However, for nanocubes, the aspect ratio will not change after the silver deposition due to its isotropic morphology. Meanwhile, the increased size of nanocubes leads to the obvious increase of the scattering intensity. Therefore, we reconstruct the electrochemical “CV” of single nanocube by taking the first-order derivative of the intensity change with time. As shown in Fig. 3e, the “CV” curve is also similar to the electrochemical CV and shows two deposition peaks. The first deposition peak may be attributed to the UPD process on the Au (100) facet as verified by the potential step experiment (Fig. 3f). Similar to Au nanooctahedrons, the negative shift of the UPD peaks for single Au nanocube (0.376 V) compared with the bulk Au (100) single crystal electrode (0.386 V) is due to the adsorbed surfactants (Fig. 3g) on NCs. The histogram of the UPD potentials of 20 individual nanocubes (Fig. 3h) reveals a broader distribution compared with the nanooctahedrons, which may indicate a better quality of {111} facet than {100} on the NCs surface. However, the onset potential of the second deposition peak (OPD) is almost the same for the two types of NCs, as in both cases the Ag is deposited over the existing Ag layer. One may argue that the first deposition peak is the preferential deposition of Ag on the vertex of NCs, since the vertex may have a higher energy than the facets. However, up to now it is still extremely challenging to see the single atomic layer deposition on the vertex for such an active metal. Instead, we simulated the scattering spectra of both Au nanooctahedrons and nanocubes before and after the deposition of Ag with different thicknesses (see Supplementary Fig. 10) to see the dependence of the spectral change during the Ag deposition on the vertex. It shows that the Ag deposits on the vertex of both NCs will lead to a red shift of the spectra, whilst on the facet a blue shift. The latter agrees with our experimental results, indicating that the first deposition peak is from the deposition on the facet rather than the vertex.

### The UPD process of Ag deposits on truncated octahedral Au NCs with two facets

**Fig. 4:**
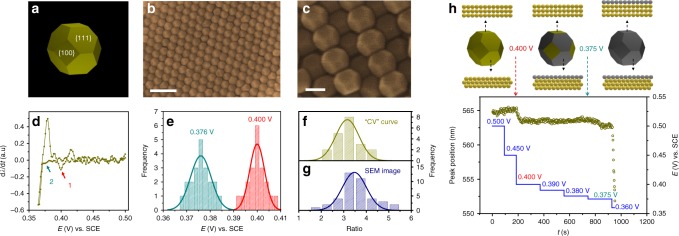
Study of Ag electrodeposit on truncated octahedral Au nanocrystals. **a** A schematic of truncated octahedral Au NC showing the structural details. SEM images of truncated octahedral Au NCs coated on the electrode obtained with **b** high and **c** low magnification, showing a highly ordered structure. **d** The optical “CV” of a single truncated octahedral Au NC reconstructed using the spectral shift of the scattering spectra at different potentials. **e** The peak potential statistics of UPD bands of {111} (red) and {100} (dark cyan) facets on the truncated octahedral Au NCs obtained from the optical “CV”s of 20 individual NCs. **f**–**g** The distribution of the area ratio of {111}/{100} on different individual NCs estimated from **f** the optical “CV”s and **g** SEM image. **h** The time-dependent peak position change of the scattering spectra of a single truncated octahedral Au NC under different potential controls, along with the corresponding schematics showing UPD processes on different facets (top graphs). The scale bars are 200 nm in **b** and 50 nm in **c**.

The above studies demonstrate that applicability of our method on the NCs enclosed by only one type of facet. It will be more important if the UPD processes on NCs with different facets can also be identified. We then applied our method to study the UPD process of truncated octahedral (TO) Au NCs (Fig. 4a–c) which consist of both {111} and {100} facets. A plot of spectral peak positions and intensities with the potential for single TO NC shows that the spectral shift is about 35 mV ahead of the intensity change (Supplementary Fig. 11a). Thus we used the spectral shift to reconstruct the optical “CV” of single NCs, as shown in Fig. 4d. Interestingly, three apparent deposition peaks can be seen at 0.400, 0.374, and 0.364 V in the optical “CV”. The first and second peaks can be identified as UPD processes on the facet of {111} and {100} according to the optical “CV” of single Au nanooctahedrons and nanocubes (also confirmed by the potential step experiment, Fig. 4h), and the third peak corresponds to the OPD process. The histogram analysis shows UPD peak potentials located at 0.400 and 0.376 V for {111} and {100} facets (Fig. 4e), respectively. The potentials are 0.397 V for nanooctahedrons and 0.378 V for nanocubes, which indicates a possible charge transfer between the neighboring facets. The minor difference in the UPD potentials of different facets on single NCs may allow us to selectively modify over a certain facet with foreign atoms on purpose to achieve the special electronic property and chemical activity.

We further calculated the area ratios of {111} to {100} using the spectral shift of Peak 1 to Peak 2 in Fig. 4d (calculation details can be found in Supplementary Note 5). The ratios are plotted with the number of NCs, which is fit with the Gaussian function (Fig. 4f). The obtained facet area ratio of 3.15 is surprisingly close to the 3.45 obtained by SEM (Fig. 4g). With the known facet ratio and particle size, the facet areas can be quantified at the single NP level with a high accuracy by EC-DFS free from the interference of the double layer charging and oxygen reduction reaction (see the obscured CV of assembled TO Au NCs in Supplementary Fig. 11b). This method can be potentially used for characterizing the NCs with multiple complex facets (such as the nanorod^27^ or bipyramid^28^) and fast screening the structure of catalyst, which is still very challenging for other methods like electron microscopy (SEM and TEM).

## Discussion

We have designed a highly sensitive EC-DFS technique allowing us to track small spectral variation during the UPD process at the single NP level. The results indicate that the EC-DFS technique is sensitive enough for direct observation of monolayer atoms deposited on different facets of single NCs under the electrochemical environment. Moreover, the spectral change can be used for qualitatively reconstructing the optical “CV” curve of single NC, which even allows the accurate quantification of the facet area of NCs. From the optical “CV” curve, the UPD potential and peak width of every single NC can be precisely obtained, which is impossible to obtain in conventional ensemble measurements but is extremely important to understand and precisely control the UPD on NCs. We believe the highly sensitive EC-DFS technique offers itself a powerful tool for studying the surface chemistry at the atomic level. If this method can be further combined with a wide field imaging technique^29–31^, it may allow characterization of the activity and structure of NCs in a high throughput way.

## Methods

### Preparation of Au NCs

Three types of Au NCs were all synthesized by the wet chemical method with the same procedure and surfactant to ensure the only variable is the facet^32^. The procedure started with the synthesis of the seeds by adding 10 ml of 0.1 M CTAB and 103 μl of 1% HAuCl_4_ in the flask followed by stirring for 5 min while keeping the temperature at 30 °C. Then, 0.6 ml of ice-cooled 0.01 M NaBH_4_ was rapidly added into the flask. The color of the solution turned from yellow to dark brown. The seed was kept undisturbed for 2 h, then 1 ml of seed sol was diluted to 100 ml with the ultrapure water for further use.

The growth solution was prepared by mixing 4 ml of 0.1 M CTAB, 1.5 ml of 0.1 M ascorbic acid, and 19.5 ml of ultrapure water into a colorimetric tube. After the solution has been stirred for 10 min at 30 °C, 41.2 μl of 1% HAuCl_4_ was added into the tube, and the solution were further stirred for 5 min. Then, 0.3 ml of diluted seed sol was rapidly added into the growth solution while stirring, and the solution was kept undisturbed at 30 °C for 12 h to obtain the seed sol of small octahedral Au NCs for the final step.

Twenty microliters of 1% HAuCl_4_ was added rapidly into the 8 ml octahedral Au seed sol at 30 °C while stirring, and the mixture was left for 1 h to ensure the completion of the reaction. The procedure was repeated for seven times to obtain the octahedral Au NCs of 50 nm. The volume of 1% HAuCl_4_ is 50 μl to obtain the truncated octahedral Au NCs of 60 nm and was repeated three times. The volume of 1% HAuCl_4_ is 150 μl to obtain the cubic Au NCs of 50 nm without repetition.

The size distribution of the three type of NCs can be found in Supplementary Fig. 8. The NCs were cleaned twice with ultrapure water before the electrochemical and spectral measurements. For the dark field measurements, the Au NCs sol were diluted 1000 times before dropped on the ITO electrode to obtain the well-dispersed sample with a large number of isolated single NCs. For the electrochemical measurements, the concentrated Au NCs sol were dropped on glassy carbon electrodes and dried in a vacuum chamber.

### Electrochemical measurements

Single crystal electrodes were fabricated following the Clavilier method^33^. The obtained single crystal beads were further polished into half-beads to obtain large exposed single crystal surfaces. The single crystal electrodes were further electrochemically polished and flame-annealed before performing the electrochemical measurements.

The NCs coated electrodes were prepared by dispersing NCs on glassy carbon electrodes. The NCs sols were first washed twice with ultrapure water by centrifugation. Afterwards, the concentrated NCs sols were dropped on glassy carbon electrodes, which were dried in a vacuum chamber. The electrochemical measurements were carried out right after the electrodes were taken out of the chamber to avoid the contamination.

The ITO electrodes were cleaned by sonication with acetone (30 min), isopropanol (30 min), ethanol (30 min), and ultrapure water (10 min, three times). A solution of 1 mM Ag_2_SO_4_ and 0.05 M H_2_SO_4_ was used as the electrolyte for Ag UPD on Au NCs and single crystal electrodes.

### Dark field scattering spectra measurements

The dark field measurements were performed on a home-modified confocal Raman microscope (Renishaw Invia, UK) that is equipped with upright (Leica DM2500) and inverted (Leica DMI3000B) microscopes. The annular white light (halogen lamp, 100 W) illumination was enabled by using a dark field condenser (oil immersion, NA 1.4–1.6). The scattering signal was collected by a water immersion objective (Nikon, ×60, NA 1.0), which were either selectively imaged on a color digital camera (Q-image EXI) or further dispersed to obtain the scattering spectra with a spectral CCD (Renishaw inVia) by a switchable mirror.

## Supplementary information

Supplementary Information

## Data Availability

The data that support the findings of this study are available from the corresponding author upon reasonable request.
