# Supplementary material for: Observing atomic layer electrodeposition on single nanocrystals surface by dark field spectroscopy
Source: Nat Commun. 2020 May 20;11:2518. doi: 10.1038/s41467-020-16405-3 (PMC7239926; doi:10.1038/s41467-020-16405-3)
Supplement: Supplementary file 1 — Supplementary Information [file 41467_2020_16405_MOESM1_ESM.pdf]

Supplementary Information

Observing atomic layer electrodeposition on single nanocrystals surface by dark field spectroscopy

Hu et al.

Supplementary Note 1. A comparison of the conventional and novel setup for the electrochemical dark field scattering technique

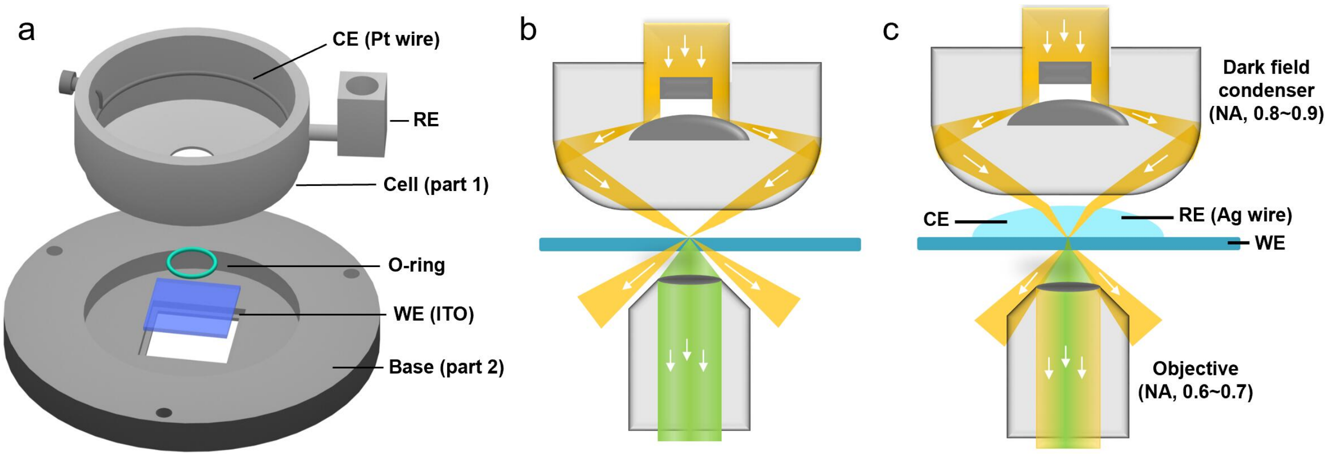

Supplementary Figure 1. A schematic of the cell and setup for the dark field imaging. a The disassembled diagram of the novel cell for the electrochemical dark field scattering technique. **b-c** The configurations of dark field setup working in air (**b**) and electrochemical environment (**c**).

Dark field imaging requires the sample illumination with high-angle annular light and signal collection at a smaller angle (Supplementary Figure 1b) to effectively avoid the influence of the transmitted light. When it is combined with the electrochemical technique, the introduction of the electrolyte will lead to a significant mismatch of the refractive index in the optical path[1]. Such a mismatch will decrease the angle of the annular light and eventually dramatically increase the background of the dark field image (Supplementary Figure 1c). To suppress the background, the conventional setup of the electrochemical dark field scattering (EC-DFS) technique normally uses a dark field condenser and collection objective with a low NA and long working distance, as shown in Supplementary Figure 1c. However, such a suppression is quite limited because it does not eliminate the optical distortion, and it also significantly decreases the collection efficiency of the scattering signal. Furthermore, most of conventional EC-DFS setup only places Pt wire (counter electrode) and Ag wire (quasi-reference electrode) on one side, which leads to the non-uniform distribution and instability of the potential over the whole electrode surface. In comparison, our setup (Figure 1a) allows the use of a water immersion objective that can effectively suppresses the refractive index mismatch and has a high collection efficiency, which can significantly increase the sensitivity of such a technique. Moreover, the cell we designed (Supplementary Figure 1a) allows us to use a Pt wire ring as the counter electrode and a commercial reference electrode of saturated calomel electrode (SCE), which provides much more stable and uniform potential control over the whole

electrode surface compared with the conventional setup. This design is especially important to UPD, because a slight potential drift may lead to obvious change in the UPD behavior.

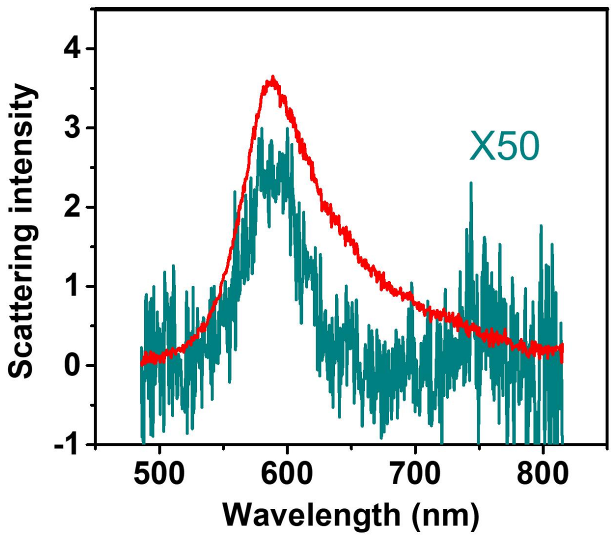

Supplementary Figure 2. A comparison of the signal to background ratio ((I_{Signal}-$I_{Background}$) / $I_{Background}$)) of the scattering spectra of single octahedral Au nanocrystal (NC) obtained by our (red curve) and conventional (dark cyan curve) setups.

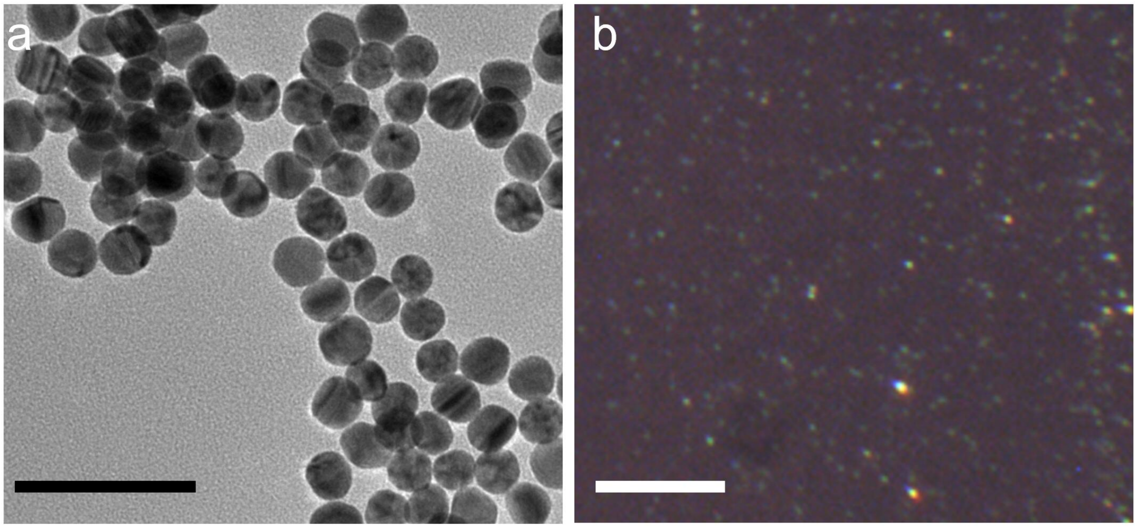

Supplementary Figure 3. Imaging small NPs with diameter of 10 – 15 nm using the novel EC-DF setup. a A TEM image of Au spherical NPs with diameter of 10 – 15 nm. **b** A dark field image of Au spherical NPs (10 – 15 nm) loaded on a quartz plate. Scale bar 50 nm in (**a**) and 10 μm in (**b**).

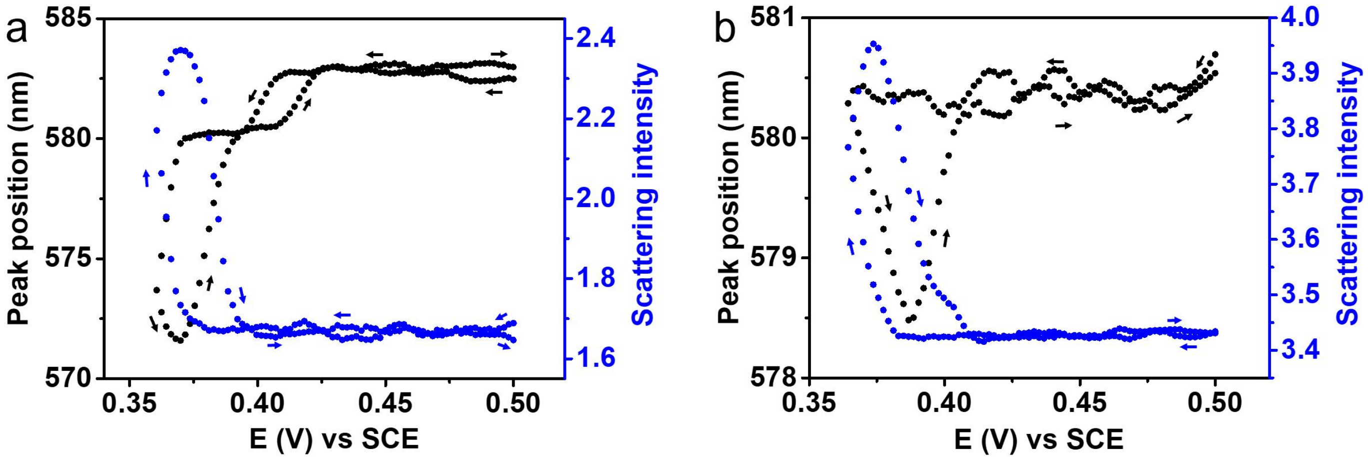

Supplementary Figure 4. The potential-dependent peak position and intensity of single Au nanoparticle scattering spectra during cyclic voltammetry: (**a**) nanooctahedron and (**b**) nanocube.

Supplementary Note 2. The relationship between the spectral variations with the Ag deposition processes

We performed several control experiments here to verify the relationship of the spectral change with the Ag deposition processes. The first one is to use the same electrolyte but free of Ag ion, while keeping all other experimental conditions unchanged. The result is shown in Supplementary Figure 5. One can see that the spectral variations was not observed over in the whole potential window between 0.500 V and 0.360 V. It demonstrates that all the spectral change we observed during the cyclic voltammetry is related to Ag ion. In addition, diluting the concentration of Ag ion from 1 mM to 0.1 mM leads to negative shift of 0.059 V of deposition peaks for both Au nanooctahedron and nanocube (see Supplementary Figure 6), which agrees well with the value predicted from Nernst equation for a one-electron reaction for the electrochemical Ag reduction reaction. These results strongly demonstrate that the spectral changes we observed were mainly induced by Ag deposition.

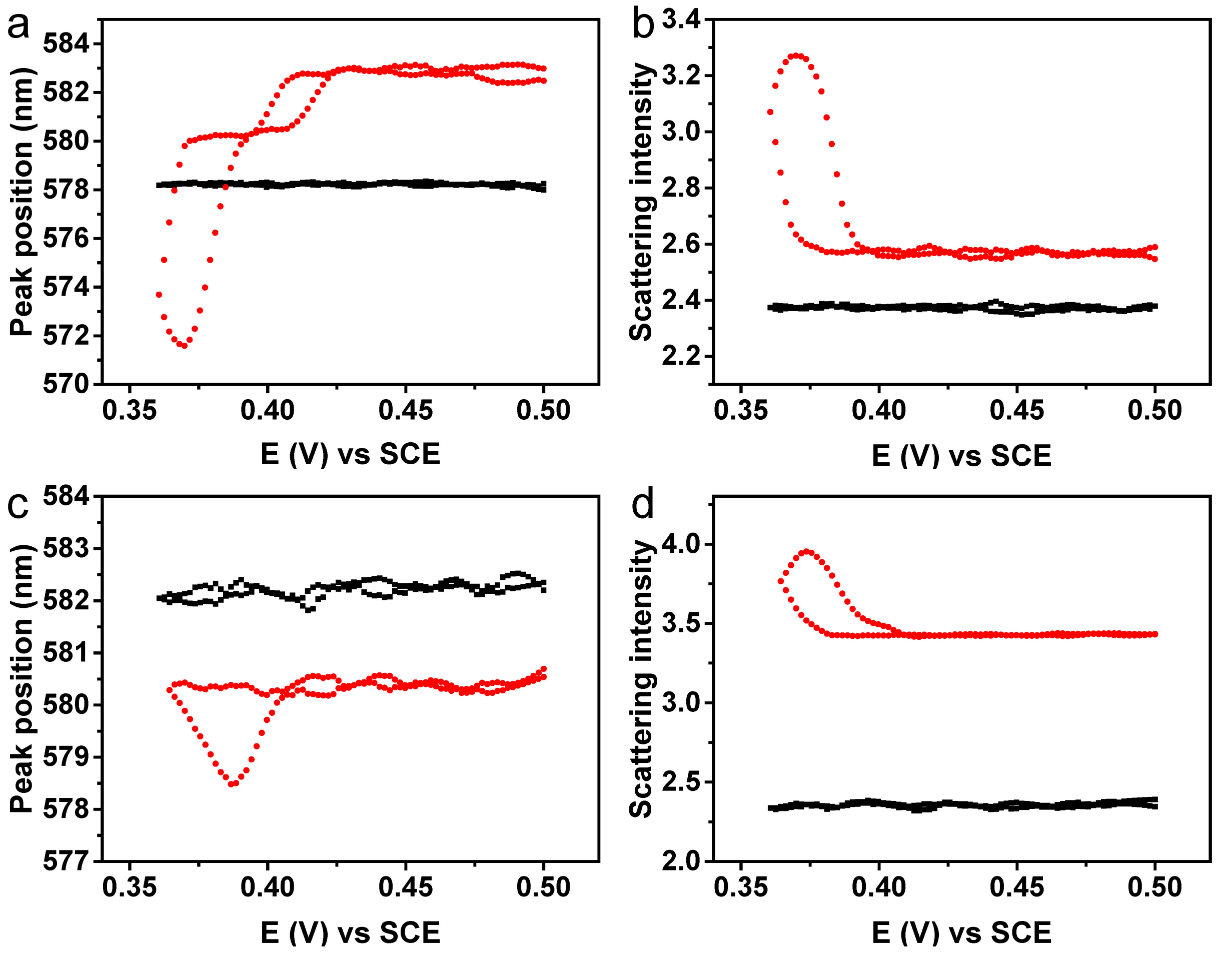

Supplementary Figure 5. The potential-dependent peak position and intensity of single Au nanoparticle spectra with (red curve) and without Ag ion (black) in the electrolyte during cyclic voltammetry: (**a**, **b**) nanooctahedron and (**c**, **d**) nanocube.

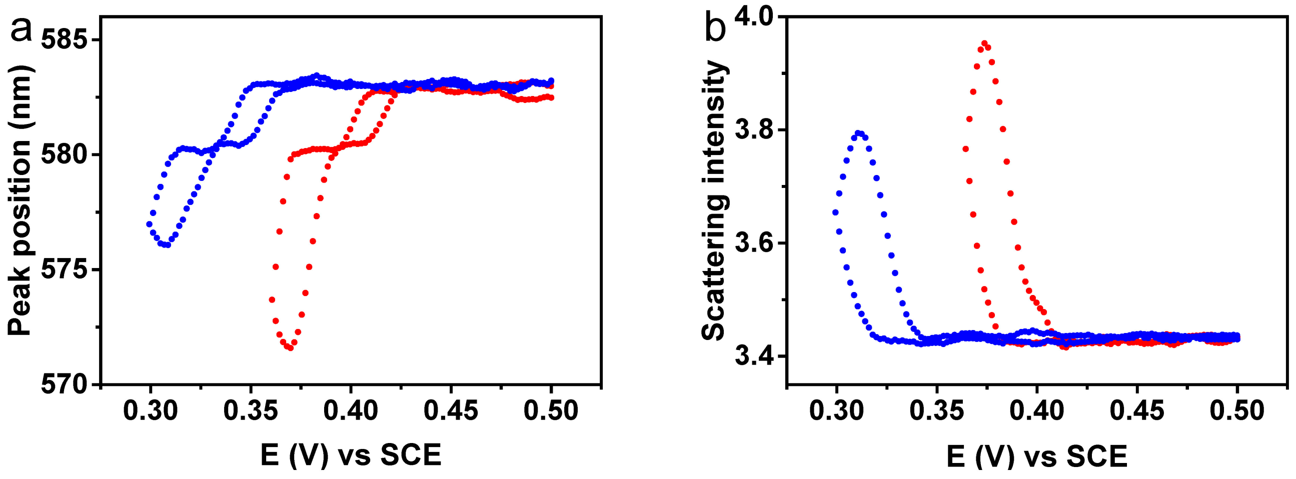

Supplementary Figure 6. The potential-dependent peak position and intensity of single Au nanoparticle spectra with 1 mM Ag_2SO_4 (red curve) and 0.1 mM Ag_2SO_4 (blue curve) during cyclic voltammetry: (**a**) nanooctahedron and (**b**) nanocube.

Supplementary Note 3. The scanning electron microscopy (SEM) characterization of Au NCs before and after the underpotential deposition (UPD) of Ag

The high energy electron beams will dramatically alter the surface state (destroying adsorbed species, creating defects in the facet, etc.) of the NCs during SEM measurements, resulting in the distortion of the UPD process. To avoid such an influence, we first obtained the SEM image (Supplementary Figure 7a and 7c, right side) of Au NCs after Ag UPD (Supplementary Figure 7b and 7d). Afterwards, the ITO electrode was taken back to the electrochemical cell and applied with a positive potential of 0.6 V for 1 min to ensure the dissolution of all the Ag atoms on the surface. Finally, the SEM characterization was performed again on this electrode for a same NC to obtain the SEM image (Supplementary Figure 7a and 7c, left side), which can be regarded as the morphology before the Ag deposition. We presented the dark field images before and after the UPD processes (see inset in Supplementary Figure 7b and 7d) as well. Unfortunately, the spectral variations are too small to be clearly observed from the dark field images.

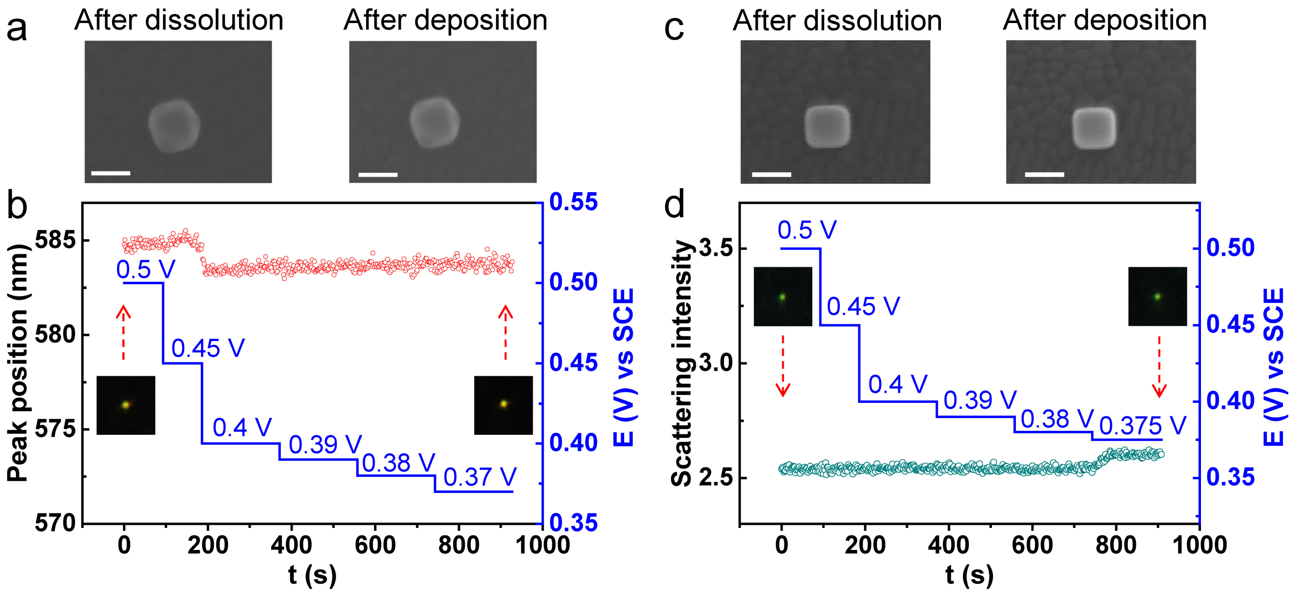

Supplementary Figure 7. SEM images of single Au (**a**) nanooctahedron and (**c**) nanocube with and without a monolayer Ag on the surface, and their corresponding potential-dependent (**b**) peak position and (**d**) intensity change during the Ag deposition to ensure the occurrence of UPD process. Scale bar 50 nm.

Supplementary Note 4. The size-dependent UPD potential distribution.

The UPD potential shows a fairly small variation (few millivolts) among different individual nanocrystals, which we believe is a result of the size-dependent effect arising from the large surface to volume ratio nature of the nanocrystals. Such an effect has been well predicted by the continuum thermodynamics[2,3], which allows us estimate the size-dependent surface energy variations by the following equation:

$$\frac{\gamma_{SV}}{\gamma_{SV0}} = 1 - \frac{S_b h}{3RD} \qquad \text{Supplementary Equation 1}$$

where γ_{SV} denotes the solid-vapor interface energy of a nanocrystal with diameter of D, γ_{SV0} (1.28 J/m^2 for Au (111)) is the surface energy of corresponding bulk crystal with same structure, S_b (106.8 J/mol.K for Au) is the bulk coherent entropy of crystals, h (0.288 nm for Au) is the covalent diameter of the bulk crystal, and R (8.314 J/mol.K) is the ideal gas constant. As shown in Supplementary Figure 8a, the size distribution of nanooctahedrons is 50 ± 9 nm. We assume the surface energy of 50 nm nanooctahedron is γ_{SV_50}, then the surface energy distribution of nanooctahedrons can be calculated as from 99.4%×γ_{SV_50} to 100.4%×γ_{SV_50} by Supplementary Equation 1, resulting in the potential variation range of 1.0%×γ_{SV_50}.

As shown in Figure 2h, the most frequent UPD potential of single nanooctahedron can be obtained as 0.397 V. Therefore, the UPD potential distribution can be estimated as 0.004 V (1.0%×0.397 V) due to the UPD potential is proportional to the surface energy. The size-dependent UPD potential variation (i.e. 4 mV) is almost equal to the band width of our experimental UPD potential statistic (i.e. 5 mV), which strongly indicates the size-dependent surface energy effect is main reason for the observed UPD potential distribution. It further demonstrates the unique sensitivity of UPD method in observing the tiny energy difference (4 meV) that is challenging to observe by other technique.

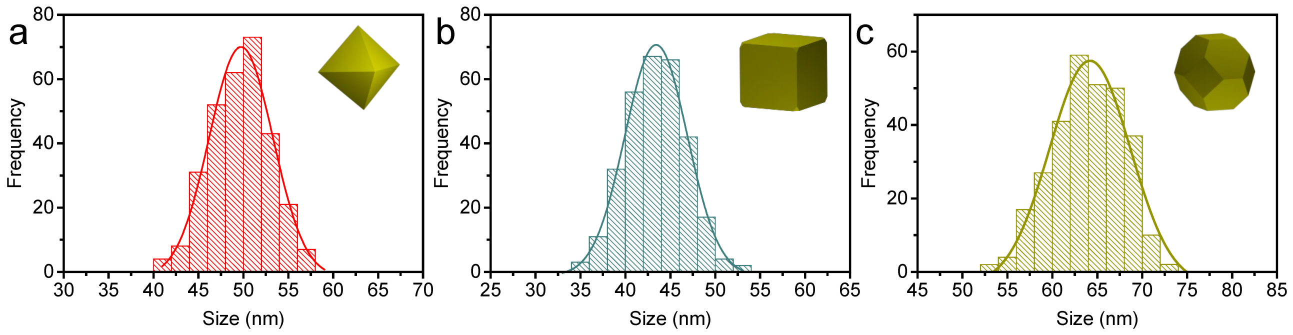

Supplementary Figure 8. The statistics of the size distribution of Au (**a**) nanooctahedron, (**b**) nanocube and (**c**) truncated nanooctahedron.

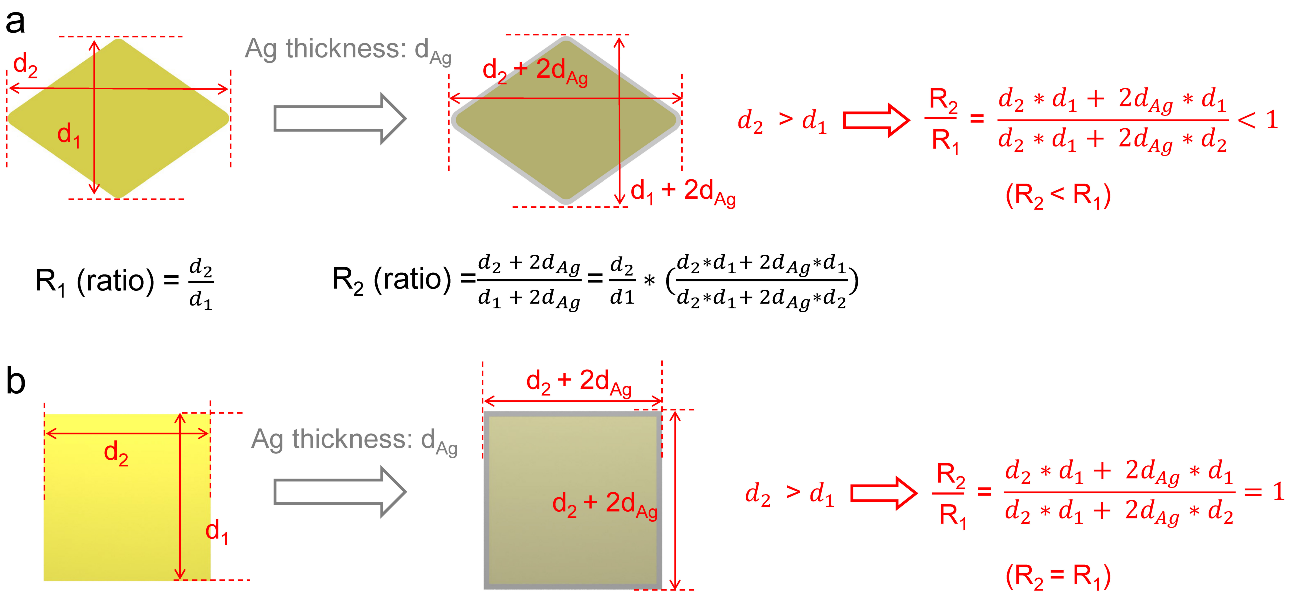

Supplementary Figure 9. The schematic and calculation of the ratio change of Au (**a**) nanooctahedron and (**b**) nanocube upon the silver underpotential deposition.

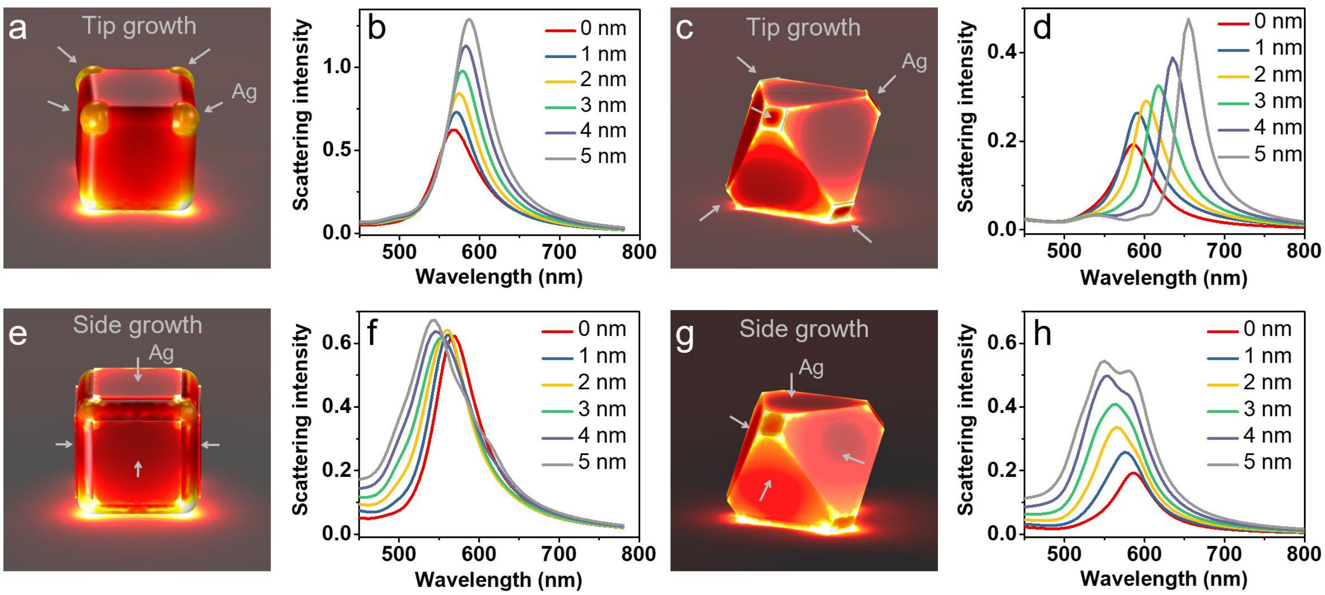

Supplementary Figure 10. Simulated electric field distribution and scattering spectra of Au NCs after Ag deposition. Electric field distributions of Au (**a**, **e**) nanocube and (**c**, **g**) nanooctahedron (excited at resonance position) with a Ag layer of 1 nm deposited on the tip (**a**, **c**) and side surface (**e**, **g**). Simulated scattering spectra of Au nanocube (**b**, **f**) and nanooctahedron (**d**, **h**) NCs with a Ag layer of 0 nm to 5 nm deposited on tip (**b**, **d**) and side surface (**f**, **h**).

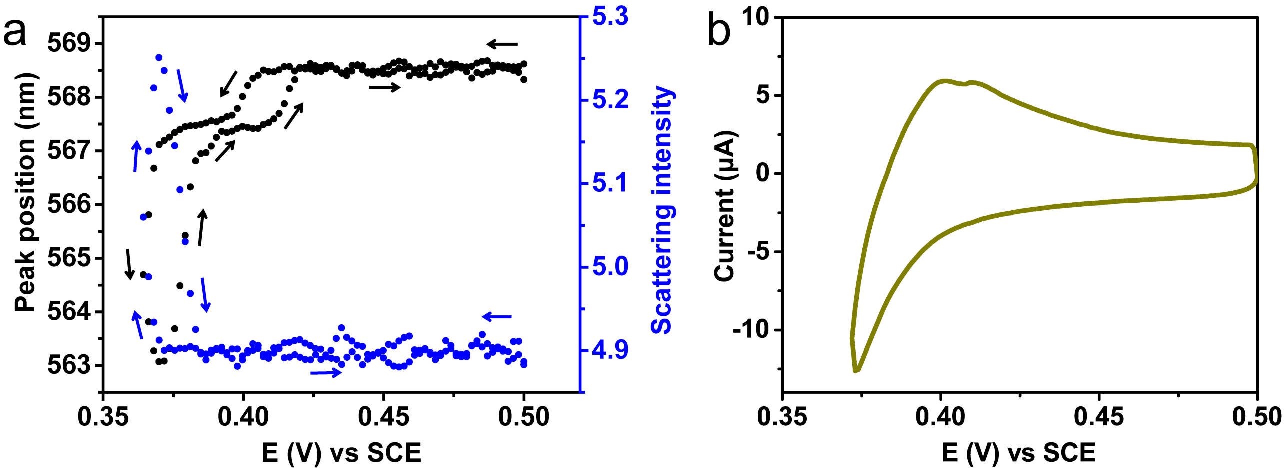

Supplementary Figure 11. a Potential-dependent peak position (black dots) and intensity (blue dots) change of single truncated octahedral Au NC. **b** CV of Ag deposition on truncated octahedral Au NCs assembled on a glassy carbon electrode in the electrolyte of 1 mM Ag_2SO_4 and 0.05 M H_2SO_4

Supplementary Note 5. Calculation of the facet area ratio of truncated octahedral Au NCs by the optical "CV" and SEM

The monolayer Ag deposited by UPD can be considered to follow the same structure as the facet below, since the lattice mismatch between Ag and Au can be negligible (~0.2 %). A truncated octahedral Au NC will expose seven {111} and six {100} facets during the electrochemical measurement according to its orientation on the electrode surface (Supplementary Figure 12). The spectral shift of two UPD peaks on different facets in the optical "CV" (Figure 4d) are proportional to the number of deposited Ag atoms. If we assume that the sensitivity of scattering spectra to the number of deposited Ag atoms are the same for the facets of Au (111) and Au (100) on a NC, we then are able to calculate the facet area ratio as follows:

$$\frac{S_{\{111\}}}{S_{\{100\}}} = \frac{\Delta\lambda_{\{111\}}}{\Delta\lambda_{\{100\}}} \times \frac{\rho_{\{100\}}}{\rho_{\{111\}}} \times \frac{6}{7} \qquad \text{Supplementary Equation 2}$$

$\rho_{\{100\}}$ and $\rho_{\{100\}}$ are Au atoms density of Au (111) and Au (100) facets (see Supplementary Figure 13). We calculated 20 individual truncated octahedral Au NCs and obtained the distribution of the facet area ratio shown in Figure 4f of main text.

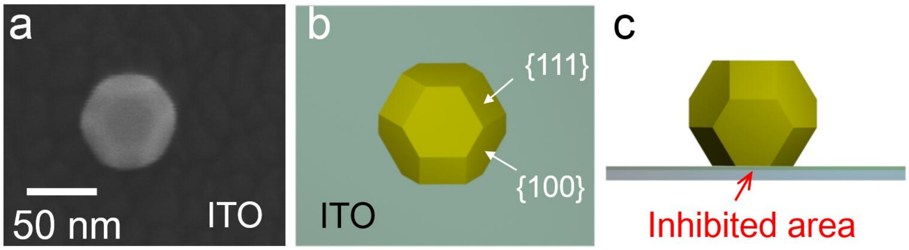

Supplementary Figure 12. a An SEM image showing the orientation of a truncated octahedral Au NC on the ITO electrode during the UPD, and its corresponding schematic of (**b**) top and (**c**) side view for showing the number of the exposed facets.

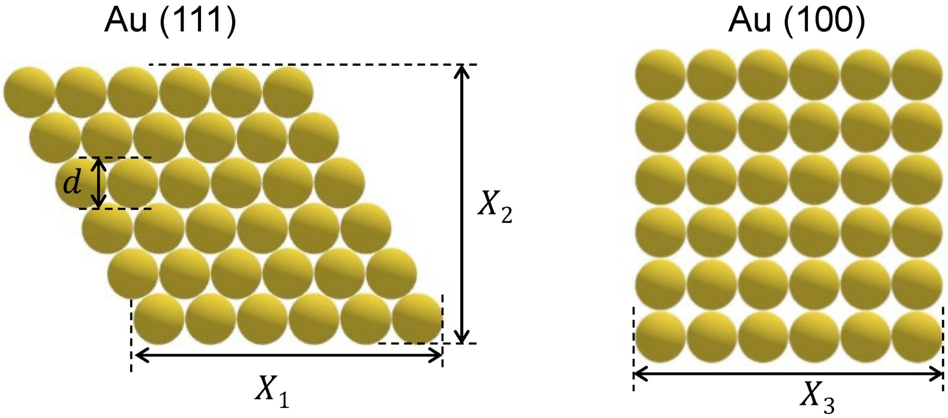

$$X_1 = 6d \quad X_2 = (\frac{5\sqrt{3}}{2} + 1)d \qquad X_3 = 6d$$

$$\rho_{\{111\}} = 36/(X_1 \times X_2) = 1.13/d^2 \qquad \rho_{\{100\}} = 36/(X_3)^2 = 1/d^2$$

Supplementary Figure 13. The schematic of Au (111) and Au (100) surface and the calculation of their corresponding Au atoms density.

To verify the accuracy of the ratio distribution obtained by the optical "CV", we did another calculation using SEM. As shown in Supplementary Figure 14, the NCs were well assembled to form a close-packed structure (side view) on the surface to minimize the measuring error induced by the variation of the orientation. As a result, we can quickly and precisely measure the length of side of the facets (L_1 and G, Supplementary Figure 14) for calculating the facet area ratio as follows:

$$\frac{S_{\{111\}}}{S_{\{100\}}} = \frac{\frac{\sqrt{3}}{4}(2L_1L_2 + (L_1 + L_2)^2)}{{L_2}^2} \qquad \text{Supplementary Equation 3}$$

The calculation of 40 individual NCs produces the ratio distribution curve in Figure 4g.

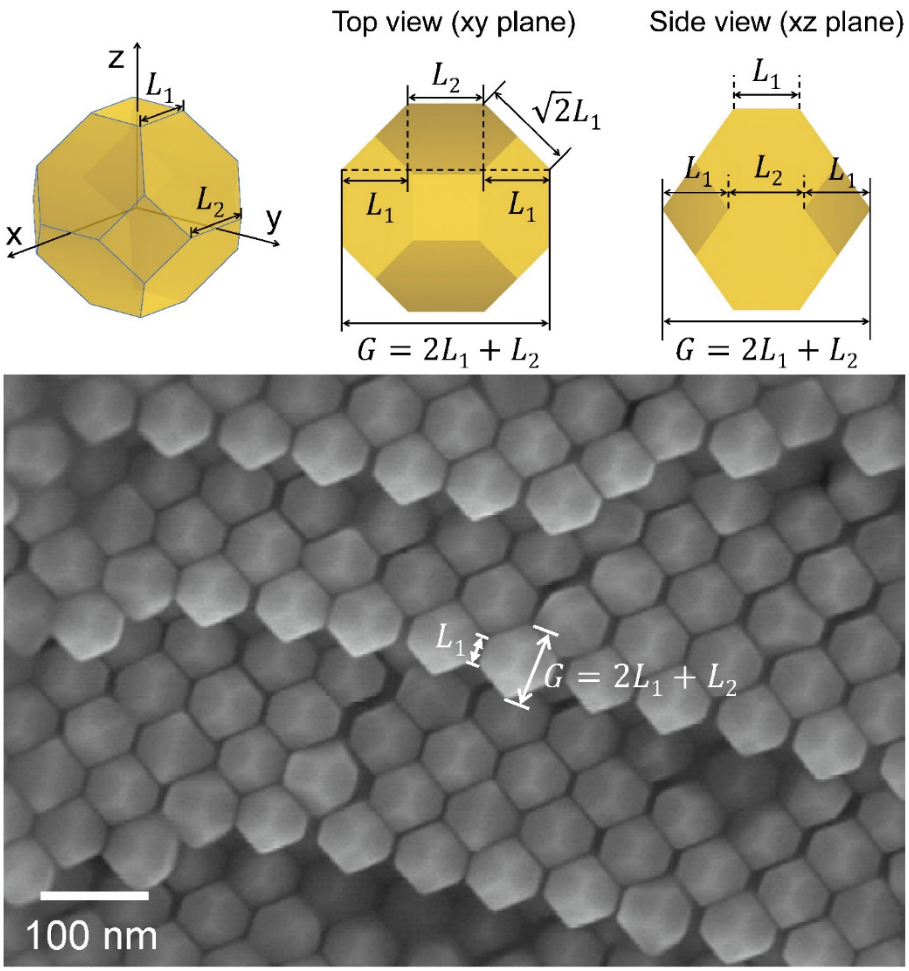

Supplementary Figure 14. The SEM image of well-assembled truncated octahedral Au NCs (side view) for calculating the facet area precisely.

We can see that calculation of the facet ratio using SEM inevitably contains the contribution from the edge of the NCs. The edge side is considered to have much higher activity and different facets, resulting in a different UPD potential, which means it will not contribute to the spectral shift of at the potentials of the Ag UPD on {111} and {100} facets. However, by SEM, we also counted the area of the edge side to {111} and {100} facets. In fact, in Supplementary Equation 3, if both L_1 and L_2 increase, the calculated area ratio will also increase. Therefore, the facet area estimated by SEM is larger than the optical "CV", which is exactly the same as our experimental results (Figure 4f and 4g). It indicates that the slightly higher peak position in the distribution curve of Figure 4g compared with Figure 4f is reasonable, and even can be used as an evidence to prove the reliability of using the optical "CV" to quantify the facet area of single NCs.

Supplementary References

1. Zeng, Z. C., *et al.* Novel electrochemical raman spectroscopy enabled by water immersion objective. *Anal. Chem.* **88**, 9381-9385 (2016).
2. Lu, H. M. and Jiang Q. Size-dependent surface energies of nanocrystals. *J. Phys. Chem. B* **108**, 5617-5619 (2004).
3. Jiang, Q., Li, J. C., and Chi, B. Q. Size-dependent cohesive energy of nanocrystals. *Chem Phys Lett* **366**, 551-554 (2002).
